# Acoustic Inversion in Optoacoustic Tomography: A Review

**DOI:** 10.2174/15734056113096660006

**Published:** 2013-11

**Authors:** Amir Rosenthal, Vasilis Ntziachristos, Daniel Razansky

**Affiliations:** 1 Institute for Biological and Medical Imaging, Helmholtz Zentrum München, Ingoldstädter Landstraße 1, Neuherberg 85764, Germay;; 2 Chair for Biological Imaging, Technische Universität München, Ismaninger Str. 22, München 81675, Germany

**Keywords:** Optoacoustic imaging, photoacoustic imaging, tomography, inverse problems, ultrasound detectors, algorithms, acoustic waves.

## Abstract

Optoacoustic tomography enables volumetric imaging with optical contrast in biological tissue at depths beyond the optical mean free path by the use of optical excitation and acoustic detection. The hybrid nature of optoacoustic tomography gives rise to two distinct inverse problems: The optical inverse problem, related to the propagation of the excitation light in tissue, and the acoustic inverse problem, which deals with the propagation and detection of the generated acoustic waves. Since the two inverse problems have different physical underpinnings and are governed by different types of equations, they are often treated independently as unrelated problems. From an imaging standpoint, the acoustic inverse problem relates to forming an image from the measured acoustic data, whereas the optical inverse problem relates to quantifying the formed image. This review focuses on the acoustic aspects of optoacoustic tomography, specifically acoustic reconstruction algorithms and imaging-system practicalities. As these two aspects are intimately linked, and no silver bullet exists in the path towards high-performance imaging, we adopt a holistic approach in our review and discuss the many links between the two aspects. Four classes of reconstruction algorithms are reviewed: time-domain (so called back-projection) formulae, frequency-domain formulae, time-reversal algorithms, and model-based algorithms. These algorithms are discussed in the context of the various acoustic detectors and detection surfaces which are commonly used in experimental studies. We further discuss the effects of non-ideal imaging scenarios on the quality of reconstruction and review methods that can mitigate these effects. Namely, we consider the cases of finite detector aperture, limited-view tomography, spatial under-sampling of the acoustic signals, and acoustic heterogeneities and losses.

## 1. INTRODUCTION

Over the past two decades, optoacoustic imaging has seen steady growth and has demonstrated notable capabilities to visualize living biological tissues with multiple applications emerging in both small-animal and clinical imaging [1-10]. Nowadays, the terms optoacoustic and photoacoustic are equally used to describe the effect of acoustic wave generation by transient light absorption. Optoacoustic imaging is based on the principles of the photophonic effect, which was first described in the late 19th century by Alexander Graham Bell and Charles Sumner Tainter [11], who recognized that sound waves can be generated through the absorption of modulated light and its conversion into heat. Optoacoustics has been used since the late 1930s for sensitive spectroscopy of gases [12]. However, lack of suitable pulse-laser technology, wideband sensitive ultrasonic detectors, and data processing capacities, have made progress and application challenging. In the 1970s, the effect was first suggested for sensing biological tissue [13]. While the physical underpinnings of optoacoustic wave generation in solids and liquids had been long established [14], first imaging studies concentrated on depth profiling of one-dimensional layered tissues [15]. With the adoption of analytical inversion formulae from the field of computerized tomography, two- and three-dimensional optoacoustic tomographies have later become possible [16]. 

In modern biomedical optoacoustics, the tissue is irradiated with nanosecond-duration pulses of light, resulting in the generation of ultrasound waves due to optical absorption and rapid thermoelastic expansion [17]. Even though additional methods of optoacoustic signal generation exist, *e.g.* using modulated continuous wave sources [18], methods relying on pulsed excitation exhibit significantly better imaging performance in terms of sensitivity [19]. For deep tissue imaging applications, optical parametric oscillators are often used to provide wavelength tunability with pulse repetition rates in the order of a few tens of Hertz and per-pulse energies in the millijoule range [8]. In optoacoustic microscopy and other superficial applications, where such high per-pulse energies are not required, other types of sources in the microjoule and nanojoule ranges are considered as well, including high repetition rate lasers [20], laser diodes [21] and fiber lasers [22]. 

Although a great variety of optoacoustic-based techniques exist for imaging and sensing of biological tissue, *e.g.* optical resolution microscopy [6] and flow cytometry [23], the focus of this review is solely on tomographic imaging scenarios, such as those found in Refs. [2, 8, 10, 24-46]. In optoacoustic tomography, the optically generated pressure profiles are subsequently captured by ultrasound detectors surrounding the imaged object. Acoustic coupling between the imaged object and the detector is usually ensured by water or coupling gel. The tomographic detection of ultrasound is performed by either scanning a single detector, or detector array, around the imaged object or alternatively using multiple detectors to simultaneously capture the generated acoustic signals. The latter configuration allows for ultrafast data acquisition, *e.g.* reconstruction of three-dimensional images from a single laser shot [24]. In comparison to the purely acoustic ultrasonography, the acoustic signals in optoacoustic tomography are generally weaker and more broadband, making their detection more challenging. Nonetheless, piezoelectric detectors, originally designed for ultrasonography, have been shown to be appropriate for optoacoustic tomography as well, and have enabled much of the progress in the field. In recent years, optical interferometric detection of ultrasound has emerged as a possible alternative to conventional piezoelectric technology, which shows promise for future miniaturization of optoacoustic imaging systems [34, 39, 47-50].

Due to its hybrid nature, optoacoustic tomography combines highly attractive features attributed to both light and sound, including rich contrast and high versatility in sensing diverse biological targets, excellent spatial resolution not compromised by light scattering, and relatively low cost of implementation. From a technical point of view, the ultimate goal of optoacoustic tomography is creating a quantified three-dimensional map of the optical absorption in tissue. In many cases, multispectral measurements may be used to extract quantified information on the distribution of tissue chromophores based on their optical absorption spectrum [51-54]. In order to achieve quantified imaging, two inverse problems must be solved: one acoustical and one optical. The acoustic inverse problem involves mapping the energy deposited in the tissue from the tomographic measurement of the acoustic signals, whereas the optical inverse problem involves turning the acoustic reconstruction into a quantified image of the optical absorption coefficient. The application of optoacoustic tomography to imaging living objects presents major challenges to solving both the optical and acoustical inverse problems. Optically, the large variations in the optical scattering and absorption coefficient of biological tissue lead to a highly complex non-linear inverse problem, whereas the processing of multi-spectral data often requires some *a priori* knowledge of the background spectrum [51] and accounting for depth-dependant variations in the light spectrum [53]. Acoustically, practical considerations in system design as well as acoustic heterogeneity and loss often lead to distorted, incomplete measurement data, which in turn may result in distorted reconstructions and imaging artifacts. 

The focus of this review paper is on the technical aspects of the *acoustic* inverse problem in optoacoustic tomography. Mathematically, the acoustic inverse problem is agnostic to the type of electromagnetic energy deposited in the tissue, and therefore in some of the work cited in this review it is studied in the context of *thermoacoustic* tomography, where the excitation is performed using microwave radiation [25, 27, 29, 32]. For a detailed review of the *optical* inverse problem and of applications of optoacoustic imaging, we refer the interested reader to Refs. [55-60] and the references contained therein. As optoacoustic tomography has grown into a highly versatile imaging technology, the acoustic inverse problem in fact represents a series of problems whose formulations depend on the specific implementation used. Practical considerations such as high sensitivity, short imaging sessions, and geometrical compatibility to the imaged object often play a decisive role in the design of optoacoustic systems. The result is often a sub-optimal acoustic measurement due to factors such as the frequency response of the acoustic detector, the finite aperture of the detector, limited-view tomography, etc. It is therefore important to realize what the effect such practical considerations have on the resulting acoustic inversion and to determine which reconstruction algorithms are most appropriate for a given scenario. Accordingly, much of the review is devoted to the relations between the theoretical and experimental facets of optoacoustic tomography. 

The paper is organized as follows: In Section 2 we discuss the forward acoustic problem in terms of both its basic mathematical description and the experimental techniques used for acoustic detection. In Section 3 we review the state of the art of acoustic inversion algorithms, which we divide to 4 categories: time-domain (so called back-projection) formulae, frequency-domain formulae, time-reversal algorithms, and model-based algorithms. In Section 4 we discuss the effect of non-ideal imaging scenarios on the characteristics of the acoustic inversion, and the conclusion is given in Section 5. 

## 2. THE FORWARD PROBLEM

In optoacoustic tomography, the acoustic forward problem relates to the calculation of the acoustic fields in time and space from a *known* heat source, or optoacoustic source., H(r,t) ., which represents the electromagnetic energy deposited in the medium per unit volume and per unit time. As discussed in the Introduction, the physical processes leading to the generation of H(r,t) may be considered as a separate physical problem, solely related to the electromagnetic (optical) properties of the medium and of the excitation sources, and are outside the scope of this review. 

In the case of acoustically homogeneous liquid medium, under the condition of thermal confinement, the optoacoustically induced pressure wave p(r,t) obeys the following differential equation [61]:

(1) $$\frac{\partial^{2}p\left(r,t\right)}{\partial t^{2}}-v^{2}\nabla^{2}p\left(r,t\right)=\Gamma \frac{\partial }{\partial t}H\left(r,t\right),$$

where v is the speed of sound in the medium, and Г is a dimensionless parameter called the Grüneisen coefficient, which describes the conversion efficiency of heat to pressure. Equation (1) is given in 3D, where r=(x,y,z). The conventional solution to Eq. 1 is based on the free-space Green’s function [62]. Briefly, replacing the right-hand side of Eq. 1 by δ(r)δ(t), where δ is the Dirac delta function, leads to the following solution:

(2) $$p_{\delta }\left(r,t\right)=\frac{\delta \left(\left|r\right|-\nu t\right)}{4\pi \left|r\right|}$$

Since Eq. (1) is linear, any solution can be represented as a superposition of the fundamental solution given in Eq. 2, *i.e.*

(3) $$p\left(r,t\right)=\frac{\Gamma }{4\pi }\int \frac{\delta \left(\left|r-r'\right|-\nu (t-t')\right)}{\left|r-r'\right|}H(r',t)dr'dt'$$

**Fig. (1):**
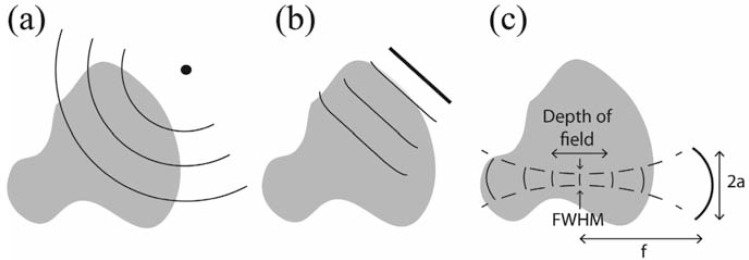
A schematic 2D description of the 3 common detector types used in optoacoustic tomography: (a) point-like detector, whose aperture is significantly smaller than any feature in the optoacoustic source; (b) line detector (either finite or infinite); and (c) focused detector. In 3D, most ultrasound detectors used in the field may be represented as a combination of these three options. The figure further illustrates the different detection patterns characterizing these three geometries, where the solid curves represent positions for which the detector is sensitive and the detection delay is constant. The case of point-like detectors is described by Eq. 4. For line detectors, if the detector is significantly longer than the imaged object, it may be approximated by an infinite line, and the detection pattern is then described by Eq. 10. In the case of focused detectors, the full-width-at-half-maximum (FWHM) and the depth of field are given by Eq. 12 and 13, respectively.

In most optoacoustic imaging applications, Eq. (3) may be simplified by recognizing that the temporal duration of the electromagnetic excitation is shorter than the temporal resolution of the acoustic detectors. In this case, the heat source may be approximated by H(r)δ(t)., where H(r)δ(t) is the deposited energy per volume, and Eq. 3 takes the form of

(4) $$p\left(r,t\right)=\frac{\Gamma }{4\pi \nu }\frac{\partial }{\partial t}\int_{\left|r-r'\right|=vt}\frac{H(r')}{\left|r-r'\right|}dr'$$

where integration is performed over a sphere, as depicted in (Fig. **1A**). 

Equation 4 offers a simple way to calculate the pressure field at a specific position and time instant and is the basis for several inversion approaches, as discussed in the following sections. Nonetheless, when one wishes to calculate the pressure fields at numerous positions, so called *k-wave techniques *are preferable [63], in which the 3D spatial Fourier transform is used. In k-space, for a heat source H(r)δ(t), Eq. 1 takes the form of

(5) $$\left(\frac{d^{2}}{dt^{2}}-v^{2}{\left|k\right|}^{2}\right)\hat{p}\left(k,t\right)=\Gamma \hat{H}(k)\frac{d}{dt}\delta (t),$$

where $\hat{p}(k.t)$ and $\hat{H}(k)$ are the spatial Fourier transforms of p(r.t) and H(r '), respectively, and $k=(k_{x},k_{y},k_{z})$ is the spatial frequency. The solution of Eq. 5 is found using the Green’s function and is given by [63]

(6) pˆ(k.t)= Г Hˆkcosvkt,

for t≥0, and p(r,t) is given by

(7) $$p(r,t)={\mathrm{Г F}}^{-1}\left\{\hat{H}\left(k\right)\cos\left(v\left|k\right|t\right)\right\},$$

where $F^{-1}$ denotes the inverse Fourier transform . In contrast to Eq. 4, Eq. 7 offers a direct way to calculate pressure field for all positions in space for a given time instant *t* in a single step. Equation 7 also shows that the initial pressure field $p_{0}\left(r\right)=p\left(r,t=0\right)$ is proportional to the optoacoustic source:

(8) $$p_{0}\left(r\right)=\mathrm{Г H}(r)$$

### 2.1. 2D Representations

Although wave propagation in optoacoustic tomography is inherently a 3D phenomenon, in some imaging geometries, 2D representations are valid. We first consider the case in which the optoacoustic source lies on the plane z=0, *i.e.*

(9) H(r)=H(x,y)δ(z)

In this case, the solution to the forward problem may be found by substituting Eq. 9 in Eq. 4. The result is the same equation only with **r** replaced by ρ=(x,y), *i.e.* the integration is performed over circles rather than spheres. This 2D representation is often used when light-sheet illumination and/or focused acoustic detectors are used [2, 7, 8, 37]. The validity of this model for specific imaging scenarios is discussed in Section 4.5. 

An additional 2D version of the optoacoustic equation is obtained when homogeneity in *z* is assumed, leading to $\mathrm{\partial p}\left(r,t\right)/\mathrm{\partial z}=0$. Equation 1 thus takes the form

(10) $$\frac{\partial^{2}p\left(\rho ,t\right)}{{\mathrm{\partial t}}^{2}}-v^{2}\left(\frac{\partial^{2}}{{\mathrm{\partial x}}^{2}}+\frac{\partial^{2}}{{\mathrm{\partial y}}^{2}}\right)p\left(\rho ,t\right)=\frac{\partial }{\mathrm{\partial t}}H\left(\rho ,t\right)$$

The solution to Eq. 10 may be readily found by repeating the procedure in Eqs. 5-7. The result is Eq. 7 with $k_{\rho }=\left(k_{x},k_{y}\right)$ substituted in place of **k**, where the Fourier transform is performed in 2D over *x* and *y*. Although it is unrealistic to assume that the optoacoustic source is homogeneous in *z*, this 2D model also applies in imaging scenarios in which the detector is homogenous in *z*. In such cases $p(\rho ,t)\;\mathrm{and}\;H\left(\rho ,t\right)$ do not represent the pressure field and optoacoustic source, but rather their respective integrals over *z* [34, 64]. 

### 2.2. Detector Properties

The acoustic inverse problem relates to the reconstruction of the heat source H(r), or alternatively the initial pressure distribution $p_{0}(r)$, from a set of pressure waves measured at multiple positions. The pressure measurements, which are performed by ultrasound detectors, do not however exactly correspond to the mathematical description given in the previous section. Specifically, realistic ultrasound detectors are characterized by a finite aperture and finite bandwidth, or temporal resolution. The detected acoustic signals can often be modeled by the following equation [31, 65]:

(11) $$p_{\det}\left(r,t\right)=h(t)*\int_{S}p\left(r,t\right)D\left(r\right)dS,$$

where *S* is the detector’s surface, D(r) is the detector’s sensitivity distribution, and h(t) is the temporal impulse response of the detector. It is often assumed that D(r) is uniform within the detector’s surface, though some detectors are inherently characterized by non-uniform sensitivity distributions [48, 66]. 

Two types of ultrasound detectors are commonly used in optoacoustic tomography: piezoelectric detectors [67, 68], in which pressure is directly transformed into voltage, and optical detectors [34, 39, 47-50], in which pressure is interferometrically detected *via* its effect on the optical path of the interrogation beam. In the former case, the temporal response of the detector is largely determined by the acoustic and electrical impedances of the piezocomposite material, and is thus often referred to as the electrical impulse response. Many piezoelectric detectors are designed to resonate at a specific acoustic frequency to increase their sensitivity, leading to a complex electrical response. The calculation of the electrical response requires exact knowledge of its design [68], which may not always be provided by the manufacturer, and it is therefore often required that the response be directly measured, *e.g.* using the techniques in Refs. [66, 69, 70]. In Ref. [70], the difference between optoacoustic techniques and pure ultrasound techniques for characterization of ultrasound detectors is discussed. In the case of optical detectors, the temporal impulse response is determined by the thickness of the detection region [39, 65] and the acoustic impedance mismatch between the optical medium in which the interrogation beam propagates and the acoustic medium (usually water) [71]. For most optical detectors, the optical medium, leading to a relatively simple temporal impulse response, which is nothing more than a low-pass operation which corresponds to the width of the detection region [65]. The major exception to this rule is optical detectors which are based on silica fibers, which are highly acoustically mismatched to water. In that case, the temporal response needs to be either measured [66], or numerically simulated [71]. We note that when complex acoustic propagation patterns exist due to acoustic impedance mismatches, *e.g.* acoustic waves guided in silica fibers [66], or angle-dependent reflection and refraction pattern which appear in piezoelectric transducers, the model used in Eq. 11 loses its validity. Such cases would require either a more elaborate model or, alternatively, measuring the spatially dependent impulse response of the detector, as discussed in Section 3.4.

Numerous aperture types have been demonstrated for optoacoustic tomography, some chosen owing to technological constraints and some due to limitations imposed by the geometry of the imaging application. Nonetheless, with but a few exceptions [72, 73], detector surfaces used in the field may be described by a combination of two out of three simple detector types in 2D space: point, line (or line segment), and focused. In other words, the 2D surface of the detector often has a separable geometry, where the geometry on each of the surface’s dimensions corresponds to one of the three simple 1D curves listed above. (Fig. **1**) shows schematically the three basic detector geometries. The figure further illustrates the different detection patterns characterizing these geometries. In the case of a point detector, the sensitivity is isotropic while the signals detected at a given time instant are assumed to originate from sources located on spheres (3D) or arcs (2D), as shown by Eq. 4. A detector may be considered as a point detector if its size is significantly smaller than the size of the features in the optoacoustic source H(r). In the case of a line detector, sources whose detection delay is the same (*i.e.* the time it takes for the acoustic signals they generate to be detected) generally lie on a line in 2D space parallel to the detector. If the detector is a finite line-segment, the sensitivity is anisotropic and generally higher for regions directly facing the detector [74]. For infinite-line detectors, the sensitivity depends only on the distance from the detector [34]. Focused detectors are used in order to limit the sensitivity to a small region. The full-width-half-maximum (FWHM) of the focal zone depends on the focal length *f*, the transducer aperture size 2α, and acoustic wavelength [67]:

(12) FWHM=1.41λf/2a

The depth of field (DOF), *i.e.* the distance over which the sensitivity field remains within the 50% of its maximum value, is given by

(13) $$\mathrm{DOF}=9.7\lambda {\left(f/2a\right)}^{2}$$

when using Eqs. (12) and (13), two points must be considered. First, these equations have been developed for focused transducers in 3D space with circular apertures (focused disks) [67]. 2*a* in this case represents the disk diameter. In the case of non-circular apertures, or detectors that are focused only along one axis, a deviation from the constant numerical factors in Eqs. (12) and (13) is expected, but not in the dependence on the physical parameters. The second point is that in optoacoustic tomography, the source does not emit acoustic waves with a single frequency and wavelength, but is rather characterized by a broad spectrum. We elaborate further on this point in Section 4.5, where the reconstruction using focused transducers is discussed.

For detectors in 3D space, the three 2D options depicted in (Fig. **1**) lead to 6 different combinations of detector types. For detectors which are point-like in both their dimensions, the most successful implementation so far has been optical [10, 39] since in optical detection the detector’s sensitivity is often independent of its surface area. In contrast, the sensitivity of piezoelectric detectors scales with their size. Accordingly, miniaturized piezoelectric detectors used for optoacoustic tomography are often significantly larger than their optical counterparts and thus may only be considered point-like for larger features in the optoacoustic source. The combination point-line relates to detectors which are very small in one dimension and are large and straight in the other. This combination enables increasing sensitivity while still maintaining a relatively compact detector design. However, the increase in sensitivity depends on the acoustic wavelength, whereas higher wavelengths are less emphasized in the detection owing to the effect of spatial averaging on the long dimension of the sensor [74]. Piezoelectric implementations of this geometry are often based on commercially available ultrasound linear arrays [75], whereas optical implementations are based on long Mach-Zehnder or Fabry-Perot interferometers [34]. In the latter case, the length of the detector may be made sufficiently large, such that it can be approximated by an infinite line. Further increase in sensitivity could potentially be achieved by using the line-line combination, *i.e.* detectors which are flat and significantly larger in both their dimensions than the typical size of the features in the optoacoustic source [29-31].

Similarly to line detectors, focused detectors may achieve higher sensitivity than point-like detectors due to their larger size. However, unlike line detectors, the sensitivity enhancement attained in the focal zone is independent of acoustic wavelength. Thus, the sensitivity achieved by a focused detector may be significantly higher than the one achieved by a flat detector of the same size when imaging objects which are considerably smaller than the detector’s size. Additionally, since the sensitivity of focused detectors is confined to a small region, it is only necessary to create the optoacoustic source within that region. Detectors which are focused in one dimension are often referred to as *cylindrically focused* detectors. In such detectors, the focal zone has a planar geometry [2, 7]. For such detectors, the second dimension may be considered as either a point [37] or a line [76]. Detectors which are focused in two dimensions are said to be *spherically focused*, and their focal zone lies on a line [3], [67]. Although focused detectors are most commonly implemented using piezoelectric technology, it has been recently shown that an optical implementation may be enabled by using a focusing mirror [38]. 

We note that the choice of a specific detector type is not trivial and is affected by both technological considerations and the particular application. Large-area detectors have the advantage of higher sensitivity, but their size also inhibits their use in array configurations, which can simultaneously measure ultrasound at multiple positions and potentially reduce the imaging time. In some cases, focused detectors can mitigate the requirement for high-power lasers for creating the optoacoustic source by partially focusing the laser beam to a small region on the surface of the imaged object to better match the focal zone [3, 7]. However, the benefit from this approach diminishes as the size of the imaged object increases owing to scattering of the laser light within the object. Some geometries might be preferable in terms of sensitivity, but require more complex inversion algorithms to form an image. In some cases, *e.g.* in small animal imaging applications, imaging speed might be favored over spatial resolution in order to enable high throughput and reduce motion artifacts [24, 77], whereas in some stationary clinical applications, it may be preferable to have longer imaging sessions in order to obtain the highest resolution possible [26]. In addition to the aforementioned technical tradeoffs, it is also clear that an imaging system needs to be reliable, robust, and relatively easy to use. In light of these considerations, it is not surprising that so many detector geometries have been tested for optoacoustic tomography.

Finally, we note that the distinction we made between point and line detectors is not always absolute and may depend not only on the characteristic size of features in the imaged object, but also on the detection surface (see Sections 2.3 and 4.2). For imaging purposes, a more general definition of a point detector may be adopted based on the discussion in Sections 4.2: A detector may be considered to be a point detector if reconstruction algorithms for point detectors may be applied without distorting the features of interest in the imaged object. As discussed in Section 4.2, in some cases, *e.g.* spherical detection surface, such reconstruction properties may be achieved even when the detector is larger than the features of interest in the imaged object. 

### 2.3. Detection Surface

**Fig. (2):**
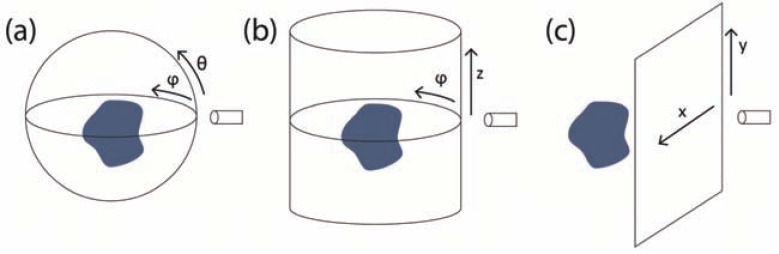
The three most common detection surfaces used in optoacoustic tomography: (a) spherical, (b) cylindrical, and (c) planar. These detection surfaces may be achieved experimentally by scanning either a single detector or a detector array over the surface. The arrows show the directions in which a single detectors needs to be scanned. The detector types appropriate for each of these detection surfaces are listed in (Table 1).

The detection surface on which the acoustic measurements are performed has a direct effect on both the quality of the reconstruction achieved as well as on the choice of reconstruction algorithms. Mathematically, if the pressure wave p(r,t) is known over a closed surface *S* that encloses the optoacoustic source, a unique solution to the inverse problem exists as well as *stable* inversion procedures [78-81]. The practical implication of this property is the following: If point-like detectors with sufficient bandwidth are placed over such a surface with sufficient density, an exact reconstruction of the optoacoustic source over any given resolution may be achieved. Three detection surfaces are of special importance in optoacoustic tomography due to their mathematical properties as well as due to practical considerations; these are the spherical, cylindrical, and planar detection surfaces, as shown in (Fig. **2**).

The three detection geometries depicted in (Fig. **2**) are often discussed in mathematical texts since they possess analytical solutions to the acoustic inversion problem [32, 40, 82]. We note that these solutions are unique even though the cylindrical and planar surfaces, which are infinite, are not closed. Practically, these surfaces, or their truncated versions in the cylindrical and planar cases, are compatible with most of the detection technologies employed in the field. For example, piezoelectric detector arrays are commonly manufactured over flat [83, 84] or curved surfaces [8, 24, 36], and mechanical scanning is considerably easier to perform over circular or straight paths than over irregular paths which offer no apparent advantage except for special cases [85]. Although most mathematical texts focus on point-detectors, these three detection surfaces have been used with a variety of finite-size detectors. (Table **1**) summarizes the different combinations of detection surfaces and detector types which have been discussed in the literature.

## 3. RECONSTRUCTION ALGORITHMS 

The mathematical foundation for much of the early development of optoacoustic reconstruction algorithms [16, 86], whether involving approximate or exact solutions, has been known for several decades now and has been applied in the fields of ultrasonic reflectivity imaging and X-ray computerized tomography [87, 88]. Nonetheless, the large diversity of imaging scenarios which exists in optoacoustic tomography, as discussed in Sections 2 and 4, as well as its unique physical underpinnings has led to new challenges specific to optoacoustic tomography. The exponential growth in computational power as well as the emergence of new algorithms in fields such as image and signal processing have created new possibilities for more versatile reconstruction algorithms which would have been considered highly impractical less than a decade ago. It is therefore not surprising that many new image-reconstruction algorithms are still being developed. Since novel detector types [39, 49, 48, 72] and detection geometries [85] are still emerging, this trend is expected to continue. 

In the following we review the state-of-the-art in algorithms for optoacoustic reconstruction in 3D and 2D. Although numerous algorithms have been proposed to this end, most may be included in one of the following categories: closed-form time-domain (back-projection) solutions, closed-form frequency-domain solutions, numerical time reversal techniques, and numerical model-based algorithms. For the 2D inverse problem, we note that in the case of homogeneity in *z* (Eq. 10), the solution $H\left(\rho ,t\right)$ is equal to $H\left(r,t\right)$ integrated over and therefore does not constitute a full solution to the inverse problem on its own. Nonetheless, a set of recovered functions $H\left(\rho ,t\right)$ obtained at different orientations of the source may be used to recover $H\left(r,t\right)$ *via* the 2D inverse Radon transform [34]. In contrast, in case of an optoacoustic source which is assumed to be restricted to a plane (Eq. 9), recovering $H\left(\rho ,t\right)$ is equivalent to recovering $H\left(\rho ,t\right)$ for the imaged plane. Practically, the reconstruction procedure may be applied for different imaging planes in the imaged object, thus recovering the entire 3D optoacoustic source plane-by-plane without the need for additional inversion steps [7].

### 3.1. Time-domain Algorithms 

Time-domain algorithms are commonly based on projecting each of the 1D acoustic signals onto 3D space in a way that is consistent with the time-of-flight principle. The back-projection process generally involves 3 steps:

1. Pre-processing: A mathematical operation performed on each of the measured acoustic signals $p\left(r_{n},t\right)$ to form a new function, which we denote here by $b\left(r_{n},t\right)$, where ${\left\{r_{n}\right\}}_{n=1}^{N}$ are the locations of the acoustic detectors.
2. Back-projection: Each of the functions $b\left(r_{n},t\right)$ is projected onto concentric spheres in 3D space based on the following equations:
  (14) $$B_{n}\left(r\right)=b\left(r_{n},\left|r-r_{n}\right|/\nu \right)$$
  The physical interpertation of Eq. 14 is that the signal at each time instant *t* is projected to all the positions in which it could have originated, *i.e.* a sphere centered on $r_{n}$ with a radius of *vt*. Additional spatial processing, *e.g.* weighting, may be performed on .
3. Summation: Image formation by summing up all the functions calculated in Step. 2.

**Fig. (3):**
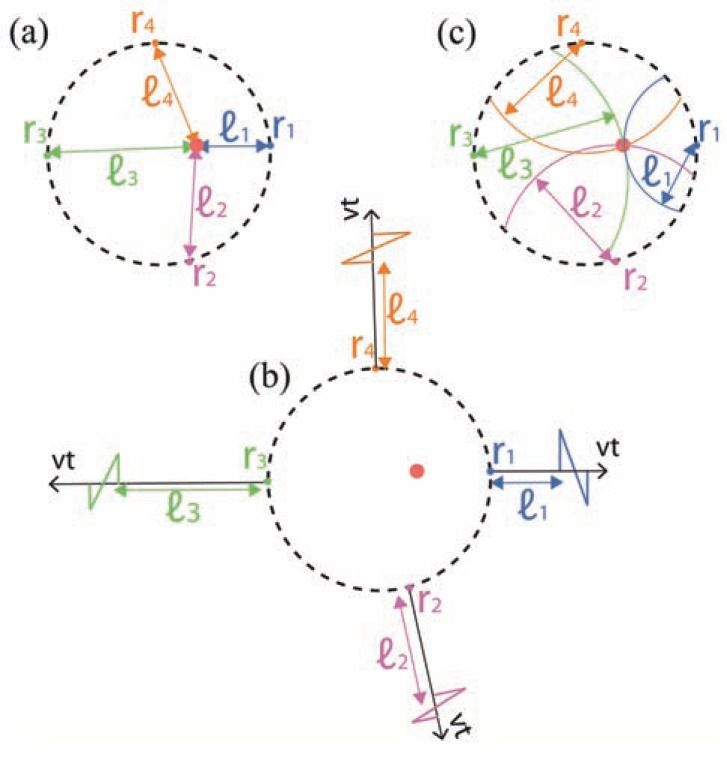
A graphical 2D illustration of the process of backprojection, discussed in Section 3.1. (a). A point source detected at 4 positions on a circular detection surface ${\left\{r_{i}\right\}}_{i=1...4}$ with the corresponding distance from the source ${\left\{\ell_{i}\right\}}_{i=1...4}$ . (b) The acoustic signals measured by the 4 detectors with delays proportional to the distances ${\left\{\ell_{i}\right\}}_{i=1...4}$ . (c) Each of the signals is projected to an arc (or spherical shell in 3D) with a radius equal to its original distance $\ell_{i}$ . The only location where all the arcs intersect is the original position of the source. Thus, the contributions of all the backprojected signals can add up coherently only at the original position of the point source. If the reconstruction formula is exact and the number of back-projected signals is sufficiently large, the originating point source will be recovered. In the case of approximate formulae, the reconstructed source may be distorted, but will still be well-localized owing to the geometrical properties of the backprojection procedure.

A geometrical representation of the back-projection procedure is given in (Fig. **3**). As shown in the figure, the success of the back-projection approach may be understood from a purely geometrical perceptive, independent of the exact functions that are back-projected. It is therefore not surprising that early applications of the back-projection concept in optoacoustic tomography achieved good reconstructions while relying on mostly heuristic reasoning [1, 89, 90], rather than rigorous mathematical analysis, which was only later developed. Arguably, the most basic implementation of the back-projection procedure is the so-called *delay-and-sum* *algorithm* in which no pre-processing is performed and $p\left(r_{n},t\right)$ are projected directly [1]. Although this approach accurately quantifies the position and size of simple optoacoustic sources, it is inadequate for quantitative imaging, as revealed by more rigorous formulations.

**Fig. (4):**
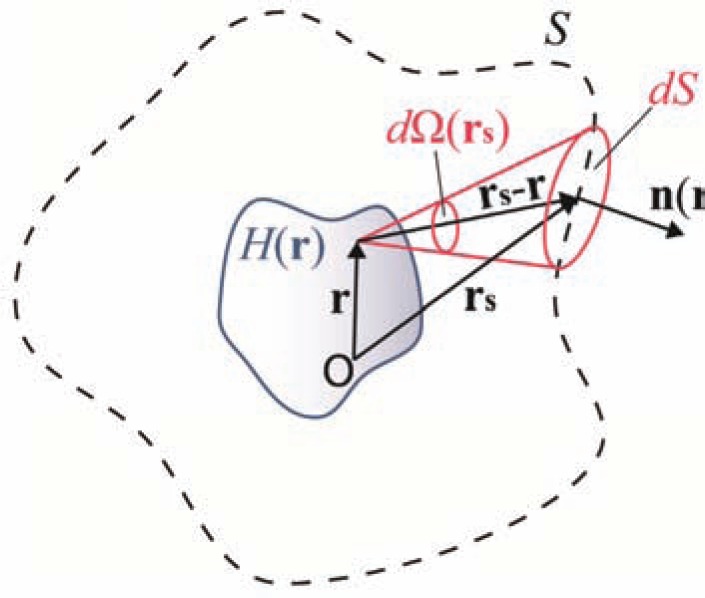
An illustration of the variables used in the back-projection formulae of Eqs. 15 and 16. An arbitrary detection surface *S* encompasses the optoacoustic source H (r). The vectors r and r_s_ represent positions in the optoacoustic source and detection surface, respectively. n_s_ (r_s_) is a unit vector orthogonal to the surface at the position r_s_, respectively. *dS* is the infinitesimal surface element and dΩ(r_s_) is the corresponding infinitesimal solid-angle element.

More advanced back-projection algorithms have been developed for the case of far-field acoustic detection [25, 33, 86]. Namely, it is assumed that the distance between the imaged object and the detectors is significantly larger than the size of the object or, alternatively, than the size of features of interest. Mathematically, the condition for the far-field approximation is that for every **r_s_** on the detection surface and every **r** within the imaged object, $\left|r_{s}-r\right|≅r_{s}$. Consequentially, the integration over the spheres in Eq. 4, may be approximated by integration over plane, leading to the 3D Radon transform. Far-field inversions have been developed in spherical, cylindrical, and planar geometries by Xu *et al.* [25] and [33]. More recently, Burgholzer *et al.* generalized the far-field inversion equation to arbitrary closed surfaces and obtained the following equation [81]:

(15) $$H\left(r\right)≅-\frac{1}{2\pi \Gamma }{\int_{S}t\frac{\partial p\left(r_{s},t\right)}{\partial t}|}_{\nu t=\left|r_{s}-r\right|}d\Omega_{r}\left(r_{s}\right)$$

where $d\Omega_{r}\left(r_{s}\right)$ is the solid angle element corresponding to the detector surface element *dS* when viewed from **r**, as depicted in (Fig. **4**). The analytical representation of $d\Omega_{r}\left(r_{s}\right)$ is given by $\frac{dS}{{\left|r_{s}-r\right|}^{2}}\left[n\left(r_{s}\right)\cdot \frac{r_{s}-r}{\left|r_{s}-r\right|}\right]$, where $n\left(r_{s}\right)$ denotes the normal vector at pointing outwards, and denotes the scalar product between two vectors. Recalling the far-field approximation, $d\Omega_{r}\left(r_{s}\right)$ may be simplified to $\frac{dS}{{\left|r_{s}\right|}^{2}}\left[n\left(r_{s}\right)\cdot \frac{r_{s}}{\left|r_{s}\right|}\right]$.

Finally, in the case of spherical, cylindrical, and planar detection surfaces, exact back-projection formulae are known. One of the most notable formulations of the back-projection algorithm is the *universal back-projection algorithm, *developed by M. Xu* et al., *which is exact for all three detection surfaces [82]:

(16) $$H\left(r\right)=\frac{2}{\Gamma }{\int_{S}\left[p\left(r_{s},t\right)-t\frac{\partial p\left(r_{s},t\right)}{\partial t}\right]|}_{\nu t=\left|r_{s}-r\right|}\frac{d\Omega_{r}\left(r_{s}\right)}{\Omega_{0}}$$

where $\Omega_{0}$ is the solid angle of the whole detection surface, and therefore is equal to 4p for spherical and cylindrical surfaces, and to 2p for planar surfaces. We note that the only difference between Eq. 16 and Eq. 15 is the addition of the term $p\left(r_{s},t\right)$ in Eq. 16, which is indeed negligible under the far-field approximation.

Despite the elegant representation of Eq. 16, its exact numerical implementation is computationally demanding, as noted in Ref. [81]. The main reason for this numerical complexity is that for each voxel in the reconstruction grid, the solid angle element has to be calculated for every discrete value of **r_s_**. For an object with M×M×M voxels and corresponding M×M back-projected signals, the result is a complexity of $O\left(M^{5}\right)$ for the trigonometric calculations involved with the calculation of $d\Omega_{r}\left(r_{s}\right)$. However, under the far-field approximation, $d\Omega_{r}\left(r_{s}\right)$ does not depend on **r** and, thus, its calculation involves only $O\left(M^{2}\right)$ operations. If the detection surface is also spherical, $d\Omega_{r}\left(r_{s}\right)$ may be approximated by the constant $dS/{\left|r_{s}\right|}^{2}$, leading to a significantly lower computational complexity. Despite the high complexity often associated with back-projection formula, recent implementations on graphical-processing-units (GPUs) [91, 92] have been shown to be highly efficient, enabling real-time reconstruction in 3D. 

In the case of 2D imaging, back-projection algorithms have been developed for plane-bound 2D sources (Eq. 9) and infinite sources (Eq. 10). In the case of plane-bound source, Filbir *el al.* demonstrated an approximate back-projection formula that may be applied to arbitrary detection geometries [93]. Formally, the reconstructed object in this algorithm is not the source, but rather a convolution of the source with a point-spread function (PSF) which approximates the delta function. In the case of infinite sources, Burgholzer *et al. *[64] demonstrated a procedure that enables applying any 3D back-projection formula to the 2D case.

One of the advantages of the back-projection approach is that it is based on robust physical principles, and therefore may produce visually pleasing results useful for identifying the underlying anatomy of the imaged specimen even when the description of the imaging scenario does not coincide with the conditions for exact reconstruction. In such non-ideal scenarios, image quality may be often improved by introducing additional weighting to the back-projected signals $b\left(r_{n},t\right)$. In Ref. [94] this concept was used for the case of limited-view tomography (see Section 4.4) to prevent over-extenuation of features for which a better angular coverage was given. In Refs. [95, 96] a more complex weighting function was used to account for acoustic heterogeneities assuming some *a priori* information on their distribution. Weighting was performed to favor signals originating close to the detectors, as they are less likely to suffer from deformation due to acoustic heterogeneities. 

### 3.2. Frequency-domain Algorithms

Frequency-domain algorithms are based on solving the inverse problem in the Fourier domain and transforming the solution back to the spatial domain. For t>0 the acoustic forward problem is described by the homogeneous wave equation. As a result, the acoustic fields may be written as an infinite sum of known product functions in four-dimensional space (r,t):

(17) $$p\left(r,t\right)=\sum_{n}a_{n}f_{1,n}\left(r_{1}\right)f_{2,n}\left(r_{2}\right)f_{3,n}\left(r_{3}\right)f_{4,n}\left(t\right),$$

where r1,r2,and  r3 are the three spatial variables that correspond to the geometry of the problem. We note that Eq. 17 is only an abstract, general representation of p(r,t), in which the sum over *n* may also correspond to integrating over a continuous parameter. The ability to transform p(r,t) into a sum of *separable* functions is at the heart of many of the frequency-domain techniques used in optoacoustic tomography. Since the base functions are pre-determined, knowing the coefficients $a_{n}$ equates to knowing p(r,t) for all values of **r** and *t*. The procedure leading to the forward solution in Eq. 7 may also be understood in the context of Eq. 17: In the forward problem, p(r,t) is known for for all values of r1,r2,and  r3. The known function p(r,t=0) can be transformed in the three-dimensional space of **r** using the same base functions as in Eq. 17:

(18) $$p\left(r,t=0\right)=\sum_{n}b_{n}f_{1,n}\left(r_{1}\right)f_{2,n}\left(r_{2}\right)f_{3,n}\left(r_{3}\right).$$

In order to compare Eqs. 17 and 18, it is required that a unique dispersion relation be known between the temporal and spatial functions, *i.e.* that each of the combinations of spatial functions in Eq. 18 corresponds to only to a single temporal function. Then, when substituting t=0 in Eq. 17 and equating to Eq. 18, one obtains

(19) $$a_{n}=\frac{b_{n}}{f_{4,n}\left(0\right)}$$

The analysis performed in Eqs. 5-7 is equivalent to the one given above with r1,r2,and r3 being the Cartesian spatial coordinates and the functions ${\left\{f_{\alpha ,n}\left(r_{\alpha }\right)\right\}}_{\alpha =1:4}$ being complex exponential functions. The calculation of $b_{n}$ from p(r,t=0) is thus performed in this case by the 3D Fourier transform. 

The procedure performed in Eqs. 18 and 19, when put in a broader context, enables calculating p(r,t) in 4D space when it is known over three of its four variables. In the forward problem, p(r,t) is known over the three spatial variables at a given time instant. In the inverse problem, p(r,t) is known over two spatial variables and over the time variable for a single value of the third spatial variable. In other words, p(r,t) is known over a surface which corresponds to the geometry used in the decomposition in Eq. 17. In both the forward and inverse problems, the degeneracy in the dispersion relation, corresponding to fields which propagate in opposite directions but have the same spatial profile, needs to be addressed by applying constraints to the problem. In the case of planar geometry [40, 97], the decomposition is to planar waves and the detection surface must also be planar. Accordingly, in the cases of cylindrical and spherical geometries [25, 32, 98], the waves are cylindrical and spherical, and the detection surfaces are a cylinder and a sphere, respectively. The planar case is favorable both in its mathematical simplicity and numerical efficiency because the decomposition in Eq. 17 may be implemented by the Fourier transform. In contrast, cylindrical and spherical waves involve complex mathematical functions, and the decomposition cannot be performed with high efficiency. We therefore limit our discussion here to the planar geometry, whereas the solutions to the cylindrical and spherical geometries may be found in Refs. [25, 32, 98]. Finally, we note that Wang *et al. *recently developed an exceptionally simple Fourier-domain formula for the spherical case that is not based on the decomposition given in Eq. 17 [99]. 

#### 3.2.1. Planar Geometry

The following analysis is based on the one given in Ref. [97], and a detailed numerical implementation of the inversion algorithm may be found in Ref. [100]. The analysis also applies to the 2D inversion problem described by Eq. 10 with only minor modifications [34]. We assume that p(r,t) is known at z=0 and wish to find H(r) or, alternatively, the equivalent initial pressure distribution p(r,t=0) (Eq. 8). According to Eqs. 6-8, the decomposition of p(r,t) into a sum of separable functions for t>0 is given by Eq. 20.

For simplicity of the analysis, we assume that Eq. (20) is valid for all values of *t*, enforcing time symmetry on p(r,t), *i.e. *.p(r,−t)=p(r,t). We denote the measurement on the plane z=0 with u(x,y,t), and its Fourier transform by $U\left(k_{x},k_{y},\omega \right)$, as shown in Eq. (21). Since u(x,y,t) is real and symmetric in *t*, its Fourier transform is symmetric in *ω*, leading to the equivalent representation given in Eq. (22). Substituting z=0 in Eq. (20), one obtains Eq. (23). Comparing Eqs. (22) and (23) one obtains the dispersion relation between *ω* and **k** and its differential, given in Eqs. (24a) and (24b). Substituting Eqs. (24a) and (24b) in Eq. (23), and comparing to Eq. (22), one obtains Eq. (25).

Equation 25 reveals that H(x,y,z) and H(x,y,−z) create the same pressure on z=0, *i.e.* planar measurements cannot determine from which side the pressure waves arrive at the plane z=0 [97]. By assuming a symmetric source, for which $P\left(k_{x},k_{y},k_{z}\right)=P\left(k_{x},k_{y},-k_{z}\right)$, one obtains

(20) $$p\left(r,t\right)={\left(\frac{1}{2\pi }\right)}^{3}\int_{-\infty }^{\infty }\int_{-\infty }^{\infty }\int_{-\infty }^{\infty }P\left(k_{x},k_{y},k_{z}\right)\cos\left(\nu \left|k\right|t\right)e^{ik_{x}x+ik_{y}y+ik_{z}z}dk_{x}dk_{y}dk_{z}$$

(21) $$u\left(x,y,t\right)={\left(\frac{1}{2\pi }\right)}^{3}\int_{-\infty }^{\infty }\int_{-\infty }^{\infty }\int_{-\infty }^{\infty }U\left(k_{x},k_{y},\omega \right)e^{ik_{x}x+ik_{y}y+i\omega t}dk_{x}dk_{y}d\omega .$$

(22) $$u\left(x,y,t\right)={\left(\frac{1}{2\pi }\right)}^{3}\int_{0}^{\infty }d\omega \int_{-\infty }^{\infty }\int_{-\infty }^{\infty }2U\left(k_{x},k_{y},\omega \right)\cos\left(\omega t\right)e^{ik_{x}x+ik_{y}y}dk_{x}dk_{y},$$

(23) $$u\left(x,y,t\right)={\left(\frac{1}{2\pi }\right)}^{3}\int_{-\infty }^{\infty }dk_{z}\int_{-\infty }^{\infty }\int_{-\infty }^{\infty }P\left(k_{x},k_{y},k_{z}\right)\cos\left(\nu \left|k\right|t\right)e^{ik_{x}x+ik_{y}y}dk_{x}dk_{y}$$

(24a) $$\omega =\nu \left|k\right|=\nu \sqrt{k_{x}^{2}+k_{y}^{2}+k_{z}^{2}}\;$$

(24b) $$d\omega =\nu^{2}\frac{k_{z}}{\omega }dk_{z}$$

(25) $$P\left(k_{x},k_{y},\sqrt{{\left(\omega /\nu \right)}^{2}-k_{x}^{2}-k_{y}^{2}}\right)+P\left(k_{x},k_{y},-\sqrt{{\left(\omega /\nu \right)}^{2}-k_{x}^{2}-k_{y}^{2}}\right)=\frac{2\nu^{2}k_{z}}{\omega }U\left(k_{x},k_{y},\omega \right)$$

(26) $$P\left(k_{x},k_{y},\pm \sqrt{{\left(\omega /\nu \right)}^{2}-k_{x}^{2}-k_{y}^{2}}\right)=\frac{\nu^{2}k_{z}}{\omega }U\left(k_{x},k_{y},\omega \right)$$

The inversion procedure may thus be summarized as follows:

1. Given the measurement data p(x,y,z=0,t) for t>0, calculate the time-symmetric function u(x,y,t)=p(x,y,z=0,t)+p(x,y,z=0,−t) and its corresponding 3D Fourier transform $U\left(k_{x},k_{y},\omega \right)$.
2. Calculate $P\left(k_{x},k_{y},k_{z}\right)$ using Eq. 26. Numerically, this step involves interpolating the data given on discrete values of *ω* to discrete values of $k_{z}$ based on the dispersion relation [100].
3. Calculate $p_{0}\left(r\right)$ by performing the 3D inverse Fourier transform on $P\left(k_{x},k_{y},k_{z}\right)$.
4. The result of Step 3 will be a source which is symmetric with respect to z=0. If it is known that the source is non-zero only for z>0, multiply the result by 2μ(z), where μ(·) is the Heaviside step function.

One of the factors that determine the quality of the reconstruction using Fourier-domain algorithm in the planar geometry is the accuracy of the interpolation performed in Step 2. To minimize image artifacts resulting from interpolation errors, regularization may be used [101]. Alternatively, Fourier-domain reconstruction may be performed without the need of regularization by using the Fourier domain synthetic aperture focusing technique (F-SAFT) [102, 103]. In F-SAFT a frequency-domain free-space propagator is used to find $P\left(k_{x},k_{y},z=\zeta ,\omega \right)$, *i.e.* the Fourier transform of the acoustic wave on the plane z=ζ, from $P\left(k_{x},k_{y},z=0,\omega \right)=u\left(k_{x},k_{y},\omega \right)$. The algorithm steps are as follows:

1. Calculate $U\left(k_{x},k_{y},\omega \right)$.
2. Propagate the fields from z=0 to all values of *z* in which the optoacoustic source is to be found using the following equation: $P\left(k_{x},k_{y},z,\omega \right)=U\left(k_{x},k_{y},\omega \right)\times \exp\left[\text{isign}(\omega )z\sqrt{{\left(\omega /\nu \right)}^{2}-k_{x}^{2}-k_{y}^{2}}\right]$
3. Integrate over frequency to find the pressure distribution at t=0: $P\left(k_{x},k_{y},z=\zeta ,t=0\right)=\int_{-\infty }^{\infty }P\left(k_{x},k_{y},z=\zeta ,\omega \right)d\omega$.
4. Perform the 2D inverse Fourier transform on $P\left(k_{x},k_{y},z,t=0\right)$ to find the initial pressure distribution p(x,y,z,t=0).

### 3.3. Time-reversal Algorithms

Time reversal generally implies that the pressure distribution p(r,t) at some t>0 may be propagated backwards in time, *e.g.* by solving Eq. 1, to achieve the initial pressure distribution and thus the optoacoustic source (Eq. 8). A trivial implementation of this concept would thus require knowing p(r,t) at a specific time instant t>0 for all positions in space. However, as the detection of the acoustic signals is performed over a surface, and not over a volume, such a trivial implementation is impractical. The first time-reversal formula compatible with the optoacoustic measurement was developed by Xu *et al.* [78] and Finch *et al.* [79] and was based on the Green’s function subjected to the Dirichlet boundary condition. Under the far-field approximation, it was shown that this formula leads to the universal back-projection formula (Eq. 16) [78]. Thus, the early analytical formulation of the time-reversal approach could be included within the back-projection framework (Section 3.1). Much of the later work on time-reversal algorithms has been based on numerical implementations [81, 104, 105], which deviated from the back-projection formalism and enabled exact reconstruction for more general imaging scenarios. Specifically, time-reversal algorithms have been demonstrated with arbitrary detection boundaries [81], acoustic heterogeneities [105, 106] and acoustic absorption and dispersion [104]. It is therefore that we consider time-reversal algorithms as a separate category of inversion algorithms. 

In the case of lossless, homogeneous acoustic medium, time-reversal is based on two properties of Eq. 1:

1. Finite time response: Since the optoaoucstic source is enclosed within the detection surface, for a sufficiently large time *T_0_*, the pressure waves generated by the source will all leave the volume trapped within the surface.
2. Invariance under time reversal: p(r,−t) fulfills the same wave equation as p(r,t).

The time reversed pressure field is defined by

(27) $$p_{tr}\left(r,t\right)=p\left(r,2T_{0}-t\right),\;T_{0}<t<2T_{0}$$

Because of the two properties described above, $p_{\mathrm{tr}}\left(r,t\right)$ fulfills Eq. 1 with the initial condition

(28) $$p_{tr}\left(r,T_{0}\right)=\left(\partial p_{tr}/\partial t\right)\left(r,T_{0}\right)=0,\;r\in V$$

And boundary values

(29) $$p_{tr}\left(r_{s},t\right)=p\left(r_{s},2T_{0}-t\right)\;r_{s}\in S,\;T_{0}<t<2T_{0}$$

where *S* is the surface over which the acoustic measurement is performed and *V* is the volume enclosed by *S*. The conditions in Eqs. 27-29 lead to a unique solution $p_{tr}\left(r_{s},t\right)$ which can be calculated numerically [81]. The time-reversed pressure field $p_{tr}\left(r_{s},t\right)$ at $t=2T_{0}$ gives the initial pressure distribution and thus the optoacoustic source. 

Generalization of the time-reversal approach for more complex acoustic media requires modifying the procedure described above. In the case of acoustic losses, the wave equation is not invariant under time reversal. Specifically, the loss term in the original equation turns into a gain term in the time-reversed equation [104]. When the medium is acoustically heterogeneous, the assumption of a finite time response may not apply as waves may be trapped in the medium owing to acoustic reverberations [105, 106]. Nonetheless, after sufficiently long time, it is still expected that the magnitude of the remaining fields in the enclosed volume becomes negligible. 

### 3.4. Model-based Algorithms

The model-based approach is based on a discrete representation of the forward acoustic problem. Namely, because the connection between the measured acoustic field and optoacoustic source is linear, its discretization may be written in a matrix form [107]:

(30) p=Mh

where **p** is a column vector representing the acoustic fields measured at a set of positions and time instants; **h** is a column vector representing the values of the energy density on the grid; and **M** is the model matrix. Once the discrete formulation has been established, the inverse problem is reduced to the algebraic problem of inverting Eq. 30. One of the main advantages of the model-based approach is the ability to include in the inversion process any linear effect in the imaging system, *e.g.* the detection field of finite-size detectors [31, 74] or acoustic heterogeneities [108-110]. 

In the ideal case of point detectors and a homogeneous lossless acoustic medium, the model matrix may be calculated by discretizing the integral relation in Eq. 4. Since the integration is performed on spherical surfaces (or arcs in the 2D case), which do not match the grid points on which the optoacoustic source is discretized, an accurate discretization of Eq. 4 requires approximating H(r) by a finite sum of *a priori* defined interpolation functions:

(31) $$H\left(r\right)\approx \sum_{n=1}^{N}h_{n}f_{n}\left(r\right)$$

where *h* are the values of H(r) on the image grid. Substituting Eq. 31 in Eq. 4, and performing the integration over $f_{n}\left(r\right)$, one obtains the model matrix **M** [31, 107, 111-113]. The accuracy of the model matrix has a direct effect on the quality of the sequential reconstruction. For example, it has been shown that discontinuities in the interpolation, *e.g.* when piece-wise constant interpolation is used, may lead to numerical errors in the reconstruction [111].

Once a model matrix for the ideal case is constructed, it may be modified to include the response of the detector. Since the response of ultrasound detectors is generally linear and time-independent, it may be described by a spatially dependent impulse response $\;R(t,r,r_{s})$, where **r** is the position of a delta-function source, and $r_{s}$ is the detector’s position on the detection surface. $\;R(t,r,r_{s})$ may be numerically simulated to include the geometrical effects of the detector’s finite aperture [68, 74] and/or experimentally measured to include the effect of acoustic and electric impedance mismatches in the detection process [69, 70]. It has been shown that the effect of $\;R(t,r,r_{s})$ may be included by modifying Eq. 4 as follows:

(32) $$p(r_{s},t)=\frac{\Gamma }{4\pi }\int |r_{s}-r'|R\left(t+\frac{\left|r_{s}-r'\right|}{\nu },r',r_{s}\right)*\frac{\delta '(|r_{s}-r'|-\nu t)}{\left|r_{s}-r'\right|}H_{r}(r')dr',$$

where * denotes temporal convolution [74]. $\;R(t,r,r_{s})$ may be either measured experimentally or calculated numerically, depending on the detector technology. When Eq. 11 is valid, a hybrid method based on both measurement and simulation may be used [114]. Since the difference between Eqs. (32) and Eq. (4) is the addition of a temporal convolution operation, the model matrix corresponding to Eq. (32) may be obtained by performing discrete convolution on the columns of the model matrix calculated for the case of ideal detectors [74].

When the inverse problem is well-posed, *i.e.* the projection data is sufficient for performing an exact reconstruction, the inversion of Eq. (30) may be performed by solving the following least-square problem [111]:

(33) $$h_{\text{sol}}=\arg\min_{h}{\left\|p-Mh\right\|}^{2},$$

where ‖⋅‖ is the $\ell_{2}$ norm. The solution to Eq. (33) is given by the Moore–Penrose pseudo-inverse:

(34) $$h_{\text{sol}}=M^{†}p,$$

where $M^{†}$ is the pseudo-inverse and is given by **M**$M^{†}={\left(M^{T}M\right)}^{-1}M^{T}$, and *T* denotes the conjugate transpose operator. The advantage of using the pseudo-inverse approach is that the pseudo-inverse matrix **M** is determined only by the experimental setup, *e.g.*, positions of the detectors, their electric and geometric responses, etc., and not by the measured data. Thus, **M** may be calculated once for a given measurement configuration with inversion reduced to multiplying it by the measured values of **p** – a process that can be realistically performed in real time. The disadvantage of the pseudo-inverse approach is that its calculation might involve multiplication and inversion of very large matrices, which may turn impractical. 

When the model matrix is too large to be inverted directly, iterative algorithms may be used instead to solve Eq. (33) [107], *e.g.* gradient descent or conjugate gradient [31]. In the iterative process, matrix-matrix multiplications are avoided, and matrix-vector multiplications are performed instead. Thus, the calculation of each iteration may be performed with high numerical efficiency. Generally, the conjugate-gradient method is a preferred method as it is characterized by a fast convergence rate. Further increase in efficiency may be achieved by using LSQR [111] – an implementation of the conjugate-gradient method which is exceptionally efficient in the case of sparse matrices. Indeed, model matrices in optoacoustic tomography are generally sparse because the acoustic signals measured at a specific projection angle and time instant are not affected by the entire image, but rather by a small portion of it, which in the case of ideal detection withers into a spherical shell. 

In many cases, the inversion problem is either ill-conditioned or ill-posed, *i.e.* the acquired projection data is insufficient to both uniquely and accurately determine the optoacoustic source over the prescribed grid. In other words, a large number of inherently different sources may all produce a dataset of acoustic signals that is very close to the measured data. In contrast to the other classes of inversion techniques discussed in this review, the model-based approach enables imposing constraints on the optoacoustic source to regularize the solution of the inverse problem. Many regularization techniques have been demonstrated for optoacoustic tomography, including Tikhonov regularization [113, 115], singular value decomposition (SVD) [115], multi-scale techniques [115, 116], limited-iteration LSQR [117], and total-variation (TV) regularization [118]. Multi-scale-based and TV regularizations rely on nonlinear optimization techniques from the field of image processing which provide statistical measures to distinguish between “natural” images and “spurious” images such as artifacts and noise [119]. 

The major downside of the model-based approach is the relatively large computational resources it requires for high-resolution imaging. This deficiency, which has traditionally limited the application of model-based techniques [61], has been mitigated in recent years with the aid of more sophisticated algorithms and better computational resources. One of the most notable hardware improvements has been the application of GPUs in model-based inversion, which was implemented for the computationally demanding TV regularization [118]. Memory requirements could be reduced by the matrix compression method [41] or on-the-fly calculation of matrix elements [31]. Fast inversion algorithms include the numerically efficient LSQR as well as the pseudo-inverse technique. Finally, it has been recently shown that the model matrix may be approximated by a set of smaller matrices using a wavelet-packet-based formalism, thus enabling the use of computationally intensive matrix-inversion algorithms [120]. 

## 4. RECONSTRUCTION CHARACTERISTICS

In this section we discuss the effect of practical limitations on the quality of reconstruction. Mathematically, the acoustic inverse problem is often described as the reconstruction of an optoacoustic source from the pressure field on a closed surface enclosing the source. In practice, the measured data is only a partial representation of the pressure field on the surface. First, as always true for experimental measurements, the projection data is contaminated by noise. Second, the bandwidth and aperture of the detector are finite, and in many cases, the approximation of a point detector is invalid. Third, the acoustic data that can be experimentally collected is always discrete whereas the exact mathematical solutions assume that the pressure field is known over continuous variables. Finally, in many cases, the imaged object is not accessible from all angles, leading to the so-called limited view scenario.

## 4.1. Detector’s Temporal Response

As discussed in Section 2.2, the measured acoustic signal may often be represented by a temporal convolution between the pressure waves on the detector’s surface and the temporal response of the detector. Much of the ultrasound detection technology used today involves resonating detectors whose response may be modeled by a bandpass filter. The loss of lower frequencies in the signal may prevent the reconstruction of the low spatial frequencies of the optoacoustic source, thus leading to an image with negative values. *Ad hoc* solutions such as the application of the Hilbert transform may lead to positive images [121], but cannot truly restore the lost low-frequency data. Although the low-frequency data in the reconstruction generally does not reveal much interesting structure, it plays a major role in the optical inverse problem of optoacoustics [122]. The loss of high frequencies in the signal leads to smearing in the detected signals and consequentially in the reconstruction.

In the case of full-view reconstruction, the effect of the detector’s temporal response on the image may be exactly quantified. Xu *et al. *showed that when the inversion operator for ideal acoustic detectors is performed on temporally convolved signals, the resulting reconstructed source would be the originating source spatially convolved with the following point spread function (PSF) [123]:

(35) $$\text{PSF=-}\frac{1}{4\pi \left|r\right|}\left[h'\left(\left|r\right|/\nu \right)+h'\left(-\left|r\right|/\nu \right)\right]$$

where h' is the derivative of the detector’s temporal response, given in Eq. (11), and |r| is the distance from the origin. A similar result has also been recently obtained by Haltmeier *et al.* [65]. An interesting property of Eq. (35) is that the PSF is isotropic. Thus, if the detected signal is smeared by the detector’s temporal response, the image will be equally smeared in all directions. 

When h(t) is known, and its corresponding spectrum is non-zero over the measurement frequency band, its effect may be theoretically eliminated by performing deconvolution. Since the PSF of Eq. 35 is spatially independent, *i.e.* every point in the reconstruction is convolved with the same PSF, the image may be deconvolved with the PSF to obtain the original image. Alternatively, the measured acoustic signal may be deconvolved with the temporal response h(t) [36], or it may be included in the model [111] when model based inversion is used. Practically, however, deconvolution may restore attenuated frequency components only when their value is above the noise level of the measurement [36]. If deconvolution is performed in order to recover frequency components for which the response is below the measurement noise floor, the result would only be amplification of the noise at these frequencies. 

## 4.2. Detector’s Aperture

Because of the finite size of the detector’s surface, the measured acoustic signal is a spatial average of the pressure field, as shown in Eq. 11. It is thus expected that when reconstruction is performed with the assumption of ideal detection, the result would be a spatially averaged (smeared) version of the originating optoacoustic source. The effect of the detector aperture on the reconstruction was studied in Ref. [123] for the spherical, cylindrical, and planar geometries. It was found that the resulting PSFs depend on the detection geometry and may be spatially dependent, *i.e.* different smearing may occur at different positions in the reconstructed source. Although the exact PSFs are not always known explicitly, their effect may be approximated in the case of small flat detectors. In Ref. [123], M. Xu *et al. *provide a simple rule to estimate the smearing in the reconstruction due to the detector’s aperture: Assuming that the detector’s surface is a disk with a diameter *δ*, if the scan is performed linearly in a certain direction, the reconstructed source will be smeared in that direction with a spatially invariant PSF with a width of ≈δ if the scan is performed over a circle with a radius $r_{0}$, the reconstructed source will be smeared tangentially with a spatially dependent PSF with a width $\approx \delta \left(r/r_{0}\right)$, where *r* is the distance of the feature of interest from the origin. In the case of a circular scan, the result is based on the assumption of a small detector $\delta \ll r_{0}$. We note that the PSFs found here are based on the assumption of an infinite detection bandwidth. As shown in Ref. [65], when accounting for both the detector’s aperture and bandwidth, the image is smeared with both the PSFs found here and in Section 4.1.

The practical implications of the PSFs on the performance of optoacoustic imaging systems are far reaching, yet are rarely discussed in the literature. When a linear scan is performed, either as part of a cylindrical scan or a planar scan, the reconstructed source will be smeared by the size of the detector, which in the case of piezoelectric technology is often in the millimeter range. Thus, sub-millimeter resolution is difficult to achieve for linear scans with standard flat detectors. In contrast, the scale of the smearing effect in circular scans may be reduced significantly below the detector’s size by increasing the scanning radius with respect to the size of the optoacoustic source. It is therefore not surprising that flat piezoelectric transducers are used in optoacoustic tomography almost exclusively with circular scans, whereas linear scans are performed with either focused detectors or miniaturized optical detectors that can achieve high sensitivity with small detector sizes (see Table **1**). 

In Ref. [61] it was noted that over-sampling in the spatial domain, *i.e.* scanning with step sizes smaller than the detector’s size, cannot mitigate the effect of the PSF due to the detector’s aperture. This assertion, however, is only true when the inversion procedure used is based on the assumption of point detectors. It has been recently shown that if the detector’s aperture is accounted for in model-based inversion, the effect of the detector’s aperture on the reconstruction may be significantly mitigated, leading to enhanced resolution [74, 124]. Alternatively, post-reconstruction processing techniques such as spin deblurring may be used to undo the effect of the detector’s PSF [125]. Nonetheless, the restoration of high frequencies in the image, which were attenuated in the measurement owing to spatial averaging, may come at a price of enhancing high-frequency noise components in the reconstruction [31]. Though regularization may reduce the effect of noise, it is clear that similarly to the case discussed in Section 4.1, resolution enhancement beyond the system’s “natural” values relies on low noise levels in the measurement. Finally, we note that the resolution enhancement reported in Ref. [74] relied on spatial over-sampling, i.e using scan steps which are smaller than the detector’s size. When over-sampling is not possible, modeling the detector’s aperture does not on its own enable resolution enhancement. 

## 4.3. Sampling

Although most mathematical formulations of the inverse problem assume that the pressure fields are known over the continuous coordinates of space and time, the measured acoustic data is inherently discrete. When designing an optoacoustic imaging system it is thus important to determine how time and space are discretized. In time, discretization is determined by the sampling rate of the acquisition of the acoustic signals, whereas in space discretization relates to the step size used in scanning. From the Nyquist sampling theorem it is known that a band-limited signal may be sampled at a rate that is equal to twice the signal’s highest frequency without loss of information. Practically, the sampling rate is often determined by some cut-off frequency above which the signal’s spectral content is sufficiently small. If the sampling rate is lower than the one prescribed by the Nyquist criterion, aliasing may occur, which may lead to false interpretation of the signal and artifacts in the reconstruction.

The case of discretization in time is straightforward: Since the signal is low-passed by the temporal response of the detector, the sampling rate should simply be chosen as twice the detector’s cut-off frequency. Determining the adequate spatial sampling rate, however, is often not trivial as it depends on detector’s aperture and on its temporal response. Generally, the effect of the detector’s finite aperture is a spatial low-pass operation on the measured acoustic signals, where the spatial coordinates are those of the detection surface (*e.g.* Fig. **2**). Assuming a flat square detector, the corresponding low-pass operation is characterized by a sinc function, which decays slowly at high frequencies. Therefore, in order to minimize aliasing, the scan step should ideally be several times smaller than the detector’s length. It has been previously noted that a practical step size is between 2 and 5 times smaller than the detector’s length [61]. In many cases, however, in order to improve acquisition speed, detector arrays are used for which the scan step is equal to the detector’s length [8, 37]. 

In Ref. [120] it was shown that the spatial frequency content of the measured acoustic signals depends on their temporal frequency content. For example, applying a *temporal* low-pass filter on the acoustic signals removes also the high *spatial* frequencies in the signals and might enable scan steps larger than the detector’s length without risk of aliasing. This relation between the temporal and spatial low-pass operations of the acoustic signals is not surprizing when considering that the PSF in Eq. 35 is isotropic and smears the reconstruction also in the smearing directions of the spatial low-pass operation. For example, let us consider the case of linear scanning with a flat detector with a width of *δ*, as described in Section 4.2. Additionally, let us assume that the acoustic signals are significantly low-passed in time, resulting in an isotropic PSF (Eq. 35) that smears the image over a scale of 10δ. Clearly, in such a case, the smearing effect due to the detector’s aperture would be negligible and would not change considerably unless the detector’s size is increased to an approximate size of 10δ or above. In other words, the result of *temporal* filtering in this case would be the loss of all *spatial* frequencies in the acoustic data above the frequency${\left(10\delta \right)}^{-1}$. The implication of this spatial low-pass filtering is that in this case the scan step may be increased above the detector’s size without risk of aliasing. 

When the acoustic signals are spatially under-sampled, streak artifacts often appear in the image [115, 116]. Numerical simulations have shown that these artifacts are most apparent when back-projection algorithms are used, whereas model-based inversion is less affected by streak artifacts [111, 113]. Within the model-based framework, it was found that the best suppression of streak artifacts may be obtained when the regularization algorithm is based on the compressed sensing approach which penalizes features in the reconstruction which are not typical to “natural” images [115, 116].

## 4.4. Limited-view Tomography

While it is preferable that the acoustic detection be performed over a closed surface, it is often not feasible because of practical constraints associated with the experimental setup and the particular imaging application. Such constraints often arise in imaging systems designed for specific organs, *e.g.* the breast [26] or skin [10], rather than in whole-body imaging scenarios [30]. When designing such systems it is important to realize how the tomographic coverage affects the reconstruction. From the discussion in Section 2.3 it is clear that the full-tomographic coverage offered by closed surfaces is not essential as infinite planar detection surfaces enable exact and stable reconstructions (see Section 2.3). 

In Ref. [126], Xu *et al. *formulated rules to determine for arbitrary surfaces the regions in space for which a full-tomographic view is effectively obtained. In these so-called *detection regions*, an accurate reconstruction may be obtained for all features in the optoacoustic source. A point is said to be in the detection region if any line that passes through it intersects with the detection surface. In other words, each point in the detection region must be covered by the detection surface over a solid angle of at least 2π steradian in the 3D case, and over an angle of π radians in the 2D case. Closed surfaces, which offer a full 4π steradian view, are thus, at least theoretically, superfluous.

**Fig. (5):**
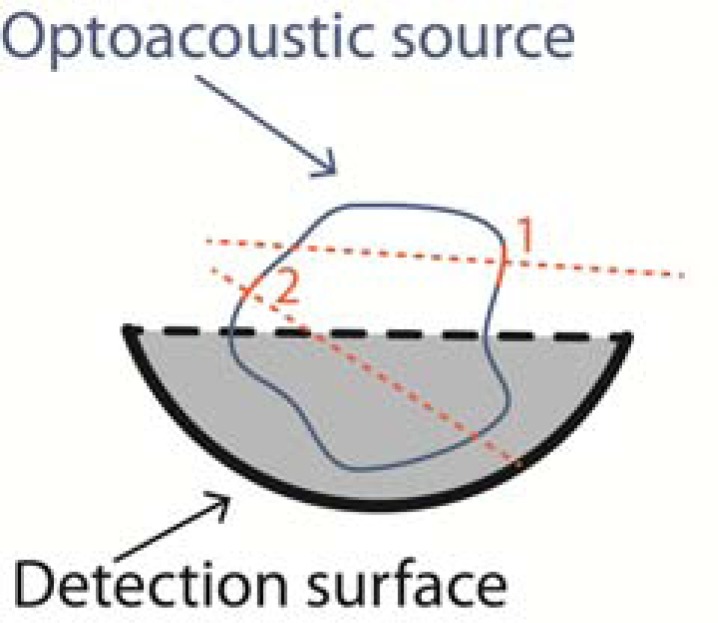
The case of limited-view detection depicted in 2D. The socalled detection region, described in Section 4.4, is marked in gray. In this region, the optoacoustic source may be exactly reconstructed. Outside the detection region, the only features which can be exactly reconstructed are those whose spatial frequencies are in the direction of the detection surface. In the case of sharp boundaries, this requirement is equivalent to having the normal to the boundary intersecting with the detection surface. Therefore, in this example, the part of the source’s boundary denoted by “1” may not be accurately reconstructed, whereas the part denoted by “2” may.

**Fig. (6):**
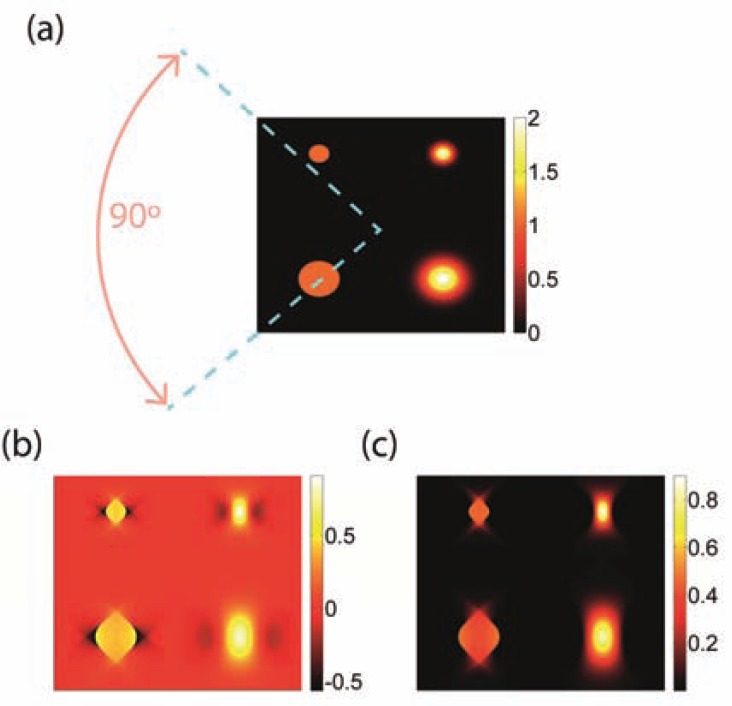
A 2D illustration of the effect of the limited-view scenario on the characteristics of the reconstruction under the far-field approximation. (a) The originating optoacoustic source and the corresponding limited angular coverage of the detection curve (or surface in 3D). (b) The image obtained by filtering out all spatial frequencies that correspond to angles outside the detection coverage. As noted in Ref. [126], limited-view detection leads to smearing of boundaries whose normal vectors are in directions not covered by the detection surface. Clearly objects which have no defined boundaries are also distorted and smeared in the direction not covered by the detection surface. As the figure shows, the smearing effect is very similar for objects of different sizes. (c) The filtered image after setting the negative values in the image to zero. The ~50% reduction in the amplitude of the objects with respect to the originating image is due to the filtering out of half the spatial frequencies.

Limited-view tomography relates to imaging scenarios in which part of the imaged object lies outside the detection region, as illustrated in (Fig. **5**). For regions outside the detection region, some features may be lost in the reconstructed source [94], [126]. Specifically, it has been noted that boundaries whose normals do not intersect the detection surface will be blurred in the reconstruction process. Thus, for the example shown in (Fig. **5**), boundary 1 will be blurred, whereas boundary 2 will be reconstructed well. Quantitative formulation of the blurring effect may be obtained by applying the wavelet-packet framework used in Ref. [120]. Although the wavelet-packet framework normally requires extensive numerical analysis, at least under the far-field approximation a simple description of the blurring effect may be obtained. In this case, the optoacoustic source may be divided into narrow bands in the spatial-frequency domain, where each band is detected only at locations on the detection surface which correspond to the direction of the frequency band. Thus, if some angles are missing in the coverage of the detection surface, approximately the same angles will be missing in the spatial-frequency spectrum of the reconstructed source. This property is more general than the one stated in Ref. [126] as it affects objects of all scales, and is not limited to only sharp boundaries, as shown in (Fig. **6**). Finally, we note that the wavelet-packet analysis does not require that the far-field approximation apply for the whole object, but rather to only small regions, as expressed by the so-called *local Radon transform* in Ref. [120]. Naturally, owing to the uncertainty principle, the size of these regions limits the resolution in which the spatial-frequencies in the object and their corresponding detection angles may be identified. 

Finally, we note that the definition of limited view is rather heuristic and is mostly useful in realistic imaging scenarios in which the detection surfaces are finite. Cylindrical and planar detection surfaces (Fig. **2B** and **2c**, respectively) technically correspond to our definition of limited-view tomography although they indeed possess stable inversion formulae. For instance, in the cylindrical detection geometry, all lines parallel to the *z* axis never intersect the detection surface, whereas for planar detection, the same is true for all the lines parallel to the detection plane. However, since all the vectors parallel to these detection surfaces cover a solid angle of 0, their effect may be neglected. Alternatively, one may adopt concepts from projective geometry, in which parallel lines are said to meet at infinity. Practically, however, cylindrical and planar detection surfaces are always finite, and thus do not have a detection region as defined in Ref. [126].

## 4.5. Focused Detectors

As can be appreciated by our review so far, most of the development of reconstruction techniques in optoacoustic tomography has focused on ideal detectors, whether infinitesimal or infinite. Model-based techniques notwithstanding, finite-aperture flat detectors are usually treated as a degenerate form of point detectors for which the point-detector approximation may still be applied in reconstruction, albeit at the price of some loss of image resolution (Section 4.2). Focused detectors represent a different class of detectors whose properties vary significantly from those of ideal detectors. As a result, most of the techniques presented in Section 3 are inapplicable to focused detectors. Focused detectors possess two properties which make them attractive for optoacoustic imaging: the ability to confine the detection field to a small region in space, and the ability to combine wideband operation with the large detection surfaces necessary for achieving high sensitivity. The combination of these two properties has made focused detectors a favorable option over flat detectors when linear scans are performed.

Optoacoustic systems in which the detection surface is cylindrical (Fig. **2.2**) are almost exclusively based on cylindrically focused detectors [2, 7, 8]. These detectors may be characterized by either a point or a line in the dimension corresponding to the circular scan and are focused in the dimension corresponding to the linear scan (see discussion in Section 2.2). The resulting focal region is thus approximately planar. Reconstruction in this case may be performed separately for each plane, *i.e.* each circular scan may be used to find an approximation for the in-plane optoacoustic source, based on Eq. 9. Since reconstructing a single slice of the optoacoustic source requires fewer projections than the entire 3D volume, cylindrically focused detectors enable fast data acquisition for *selective-plane imaging* [8]. The main disadvantage of this approach is that the thickness of the imaged plane is not constant, but is rather determined by the acoustic wavelengths or feature sizes exhibited by the optoacoustic source, as described by Eq. (12). Thus, for large objects, little or no focusing is achieved. A practical solution to the problem is high-passing the detected signals, thus limiting the reconstruction to acoustic frequencies for which the detector is focused to a sufficiently thin slice. However, as noted in Section 4.1, loss of low frequency-information in the reconstruction may hinder subsequent attempts for quantification. 

When the detection surface is planar, *i.e.* a linear scan is performed in both dimensions (Fig. **2.3**), high-resolution imaging with large-area detectors requires that the detectors be spherically focused [3]. If one ignored diffraction, and assumed ideal focusing characteristic, *i.e.* detection of only those sources lying on a line, the resulting inversion problem would be trivial, namely the integral in Eq. (4) would be replaced by the value of the optoacoustic image at a point on the detection line whose distance is directly related to the detection instant *via* the time-of-flight principle. Reconstruction would thus mean simply projecting the measured signal over the detection line. In this way, by linearly scanning the detector, the entire optoacoustic source may be reconstructed line-by-line. This procedure is extremely simple as each measurement is separately used to reconstruct a separate part of the source. In practice, however, ideal focusing along a line is impossible owing to the laws of diffraction, as described by Eq. (12) and (13). Thus, early implementations of this algorithm were limited to the focal zone of the detector, *i.e.* a cylindrical volume with the diameter and length given by Eq. (12) and (13), respectively. Although exact reconstruction formulae for focused detectors do not exist, heuristic approaches, such as the virtual detector (VD), enable expanding the volume which can be reconstructed beyond the focal-zone limit [127]. Nonetheless, the VD approach generally requires that low-frequency information be filtered out to achieve high-resolution in the reconstruction. Further improvement in reconstruction quality has been recently achieved by using a model-based approach in which the surface of cylindrically [128] and spherically [129] focused detector was included in the model. Since the model equally applies to all temporal frequencies in the detected signals, it generally enables a better reconstruction of large features in the imaged object. 

## 5. CONCLUSION

The term optoacoustic imaging represents a diverse biomedical imaging methodology capable of producing multiscale images *via* various contrast mechanisms. Much of the diversity of optoacoustic imaging stems from its hybridity, as different patterns of optical excitation and acoustic detection lead to distinct imaging scenarios. When imaging tissue at depths below the optical mean free path, optical focusing may be used to achieve high resolution, similarly to purely optical microscopy techniques. Therefore, in applications such as optical-resolution microscopy [6], the optical design has the most decisive effect on the system’s performance metrics. When imaging at depths significantly larger than the optical mean free path, the role of acoustic detection increases and becomes essential to attaining high reconstruction quality. It is in this regime, where light is fully, or almost fully, diffusive, that optoacoustic imaging may provide imaging resolutions vastly superior to purely optical techniques. Specifically, the illumination leads to the creation of acoustic sources in a large volume in the tissue, whose amplitudes are proportional to the amount of energy locally absorbed, while the spatial resolution of the reconstruction is solely determined by the acoustic, rather than the optical, characteristics of the imaging experiment. In optoacoustic tomography, a tomographic measurement of the subsequent acoustic waves provides sufficient information to retrieve a map of energy distribution in the tissue by means of acoustic inversion algorithms. As the deposited energy is proportional to the optical absorption coefficient, tomographic inversion thus yields an image with optical contrast and acoustic resolution. 

In this paper we reviewed the plurality of detection schemes (Section 2) and corresponding inversion algorithms used in optoacoustic tomography (Section 3) and discussed their characteristics. Specific attention was given to practical aspects of the techniques used in the field. Theoretically, in the case of a homogeneous acoustic medium, the optoacoustic source may be exactly recovered when the pressure waves it emits are detected over a surface enclosing the source. Numerous inversion methods, both analytical and numerical, have been developed for this ideal picture of optoacoustic tomography. Practically, the measured acoustic data is inherently limited and corresponds to only an approximate representation of the pressure waves. The consequence of this limitation is an inevitable degradation in reconstruction quality. The effects non-ideal detection patterns have on the reconstruction are largely known and are discussed in Section 4. Considerable progress has been made in mitigating these effects by means of improved hardware that better emulates the ideal detection scenario as well as new inversion algorithms that take the non-ideal detection patterns into account. Effects of non-ideal wave propagation due to acoustic heterogeneity, losses, and dispersion, are more challenging to overcome as they cannot be solved by means of better hardware, and their modeling generally requires some additional *a priori* information on the imaged object. 

In order to simplify the discussion on the numerous inversion techniques which have been developed for optoacoustic tomography, we divided them into 4 categories: time-domain (back-projection) algorithms, frequency-domain algorithms, time-reversal algorithms, and model-based algorithms. The first two categories largely involve closed-form solutions to the inversion problem. Arguably, the reconstruction approach most favored in experimental works is the back-projection approach as it is very easy to implement and is generally acceptable for most practical imaging scenarios, even when not exact. Specifically, in optoacoustic systems which rely on piezoelectric detectors, the distance of the detector from the imaged object is often sufficiently large so that the far-field approximation has some validity, and thus also Eq. 15. Fourier-domain techniques are mostly used for planar detection surfaces owing to their high numerical efficiency in that case. For spherical and cylindrical surfaces, this approach is commonly avoided due to its high mathematical and numerical complexity and the availability of the universal back-projection formula (Eq. 16), which applies to these cases. Nonetheless, the simple structure of the Fourier-domain formula recently developed in Ref. [99] for the spherical case could potentially offer an attractive alternative to the back-projection approach. 

The last two categories of inversion algorithms are largely numerical, and as such offer a more versatile solution to the inverse problem. Time-reversal techniques are based on back-tracking the propagation of the acoustic waves. Mathematically, the time axis is reversed in the differential equation describing the system, and the measured pressure distribution is used as the initial value. The solution is thus found by simply solving the new differential equation to recover the initial pressure distribution. Because the equation may be solved for any closed surface, this approach is applicable to arbitrary closed detection surfaces. Additionally, any effects that can be accurately included in the differential equation, *e.g.* frequency-dependent acoustic losses or dispersion, may be accounted for in the inversion process. This is currently an impossible feat for analytical approaches, which rely on an ideal description of the wave equation. 

Model-based algorithms represent the most general category of algorithms of those reviewed in this paper. Fundamentally, the model-based approach requires only that the tomographic problem be linear, *i.e.* that a linear relation exist between the optoacoustic source and the measured pressure fields. This relation is discretized and represented by a matrix equation, which is subsequently inverted. The advantage of this approach is that any linear effect in the system may be considered, whether it relates to the pressure wave propagation or to the acoustic detection. Thus, finite detection apertures may be taken into account in the inversion process, or more generally, any spatio-temporal detection response as long as it can be modeled or measured. In contrast, time-reversal algorithms can account for physical effects in the wave propagation, but not for effects in the acoustic detection. The major downside of model-based algorithms is the extremely large matrix sizes which are required for high-resolution imaging: ranging from several gigabytes in the 2D case to hundreds of gigabytes or more in the 3D case. Handling and processing such large data is one of the big challenges this category of algorithms is faced with. Nonetheless, the continual growth in computation power and computer memory alongside with the development of numerically efficient algorithms has already made model-based inversion a viable option for the 2D case, and an acceptable option for 3D reconstructions when high throughput is not required. The ability of model-based algorithms to account for all linear effects in the imaging systems as well as the ability to apply sophisticated regularization algorithms has been shown to increase imaging performance beyond the one achieved by classical time- and Fourier-domain techniques. As computational capacities continue to increase, one should expect to see the model-based approach applied more often in quantified experimental imaging studies, especially in non-ideal imaging scenarios where analytical inverse formulations significantly deviate from reality. 

## Figures and Tables

**Table 1.** A Review of the Combinations of Detection Surfaces and Detector Shapes Used in Optoacoustic Tomography. We Note that the Distinction Between Point and Line Detectors is not Absolute and Depends on the Feature Sizes in the Imaged Object and Possibly on the Detection Surface, as Discussed in Section 4.2.

	Spherical	Cylindrical	Planar
Point	[25], [26]	[32], [33]	[10], [39], [40]
Point-line	[27],[28]	[34]	
Line-line (flat)	[29]-[31]	[35]	[41]
Cylindrically focused		[2], [7],[8], [36]-[38]	
Spherically focused			[42], [43]
